# Identification of different *Escherichia coli* pathotypes in north and north-west provinces of Iran

**Published:** 2017-02

**Authors:** Seyedeh Tina Miri, Amir Dashti, Saeid Mostaan, Farzaneh Kazemi, Saeid Bouzari

**Affiliations:** Molecular Biology Department, Pasteur Institute of Iran, Tehran, Iran

**Keywords:** *Escherichia coli*, Pathotypes, Diarrhea, Iran

## Abstract

**Background and Objectives:**

Diarrhea is one of the most prevalent diseases in the world, specially in developing countries. One of the most important causative agents of bacterial diarrhea is diarrheagenic *Escherichia coli* (DEC) which causes gastroenteritis and this group involving enterotoxigenic *E. coli* (ETEC), enteropathogenic *E. coli* (EPEC), enteroaggregative *E. coli* (EAEC), enterohemoragic *E. coli* (EHEC), enteroinvasive *E. coli* (EIEC), diffusely adherence *E. coli* (DAEC). The aim of this study was to identify different *E. coli* pathotypes in north and north-west of Iran, among the clinical isolates.

**Materials and Methods:**

In this study for identification of *E. coli*, 170 fecal samples were cultured on MacConkey agar and identified by biochemical tests. Samples with *E. coli* characteristics were selected (145 samples) and their genomes were purified by phenol-chloroform method. After extraction of genomes, *lt* and *sta* genes identified by PCR for ETEC, *eae* gene for atypical and *eae* and *bfp* for typical EPEC, *AA* region for EAEC, *stx_1_* and *stx_2_* and *eae* genes for EHEC (*stx_1_* or *stx_2_* or both for STEC) and *invE* for EIEC.

**Results:**

Finally 10 samples identified as ETEC (%5.88), 18 (%10.58) EPEC, 6 (%3.52) EHEC and 12 (7.05%) samples were STEC. None of the samples were positive for EAEC and EIEC.

**Conclusion:**

The results obtained in this study showed that ETEC, EPEC, EHEC and STEC are prevalent bacterial agents in north and north-west of Iran. Complementary studies to identify these pathotypes in other seasons can help to adopt necessary policies against outbreaks in Iran.

## INTRODUCTION

Diarrhea is one of the prevalent diseases in the world, especially in developing countries (Asia, Middle East, Africa and Latin America) (1). According to World Health Organization (WHO), diarrhea is defined as the passage of three or more loose or liquid stools per day. The bacterial, viral, and protozoan pathogens causing diarrhoeal disease are primarily transmitted via the faecal-oral route, through the consumption of faecally contaminated food and water (2). It is estimated that there are about 2.5 million child deaths per year due to diarrhea in Iran (3). One of the most important bacterial agents which causes diarrhea is diarrheagenic *E. coli* (DEC) which causes gastroenteritis. There are six DEC pathotypes including enterotoxigenic *E. coli* (ETEC), enteropathogenic *E. coli* (EPEC), enteroaggregative *E. coli* (EAEC), enterohemoragic *E. coli* (EHEC), enteroinvasive *E. coli* (EIEC), diffusely adherence *E. coli* (DAEC). Three first groups affect small intestine and three second groups affect colon (4).

ETEC is one of the important bacterial agents which cause diarrhea and death in developing countries and travelers to tropical zones. Clinical symptoms of ETEC are like *Vibrio cholerae* but milder. Specific virulence factors like enterotoxins depart ETECs from other DECs. This pathotype is able to produce at least *lt_1_* and *lt_2_* or *sta* and *stb* toxins. *lt_2_* and *stb* don’t relate to human diseases (5–7).

EPEC is another important pathogroup of DECs which has been link to children diarrhea in developing world. Typical EPEC strains possess a plasmid called the EAF (EPEC adherence factor) plasmid. This plasmid encodes a type IV pilus called the bundle-forming pilus (*bfp*). Many types of EPEC have the *eae* chromosomal gene, encoding the outer membrane protein intimin, and elicit attaching and effacing lesions on the intestinal mucosa (6, 7, 8).

EAEC causes diarrhea in traveler children and adults in both developing and developed countries. This pathogroup define as a new intestinal pathogen and cause several outbreaks worldwide. EAEC strains adhere to HEp-2 cells and intestinal mucosa by virtue of fimbria structure known as aggregative adherence fimbria (AAFs) which it’s expression encoded by *aggR* gene. *aggR* gene is located in the main virulence plasmids of typical EAEC (p*AA*) (4, 9).

EHEC causes bloody or non-bloody diarrhea and hemolytic uremic syndrome (HUS). A wide variety of food items have been associated with disease. The key virulence factor of EHEC is Shiga toxins (*stx* genes) which is also known as Verotoxin (*Vtx*) and contains two subgroups *stx_1_* and *stx_2_*. EHEC strains of O157:H7 serotypes are the most important serotype of this group. EHEC O157:H7 is believed to have evolved from locus of enterocyte effacement-containing (LEE-containing) O55 EPEC strains that acquired bacteriophage encoding *stx*. Most of these serotypes do not contain LEE pathogenicity island. This has led to the use of Shiga toxin-producing *E. coli* (STEC) (6, 10).

EIEC is a pathotype which causes an invasive inflammatory colitis and occasionally dysentery, but in most cases, it elicits watery diarrhea and important cause of morbidity and mortality among children and adults in developing countries. Pathogenesis and especially the ability to invade the epithelium of the colon is dependent on a 220kb plasmid, called *p*Inv, which carries the genes for a type III secretion system that is used as the virulence factor. EIEC strains are very similar to *Shigella* strains (11, 12).

The aim of this study was to identify ETEC, EPEC, EAEC, EHEC (& STEC) and EIEC pathotypes in north and north-west of Iran in winter and spring seasons.

## MATERIALS AND METHODS

### Culture

In this study 170 samples were collected from different hospitals and health centers of East-Azerbaijan, Gilan, Kurdistan, Tehran, Hamedan and Zanjan between December 2012 and Juan 2013 (Table 1). Stool samples were cultured on MacConkey agar and EMB and samples with *E. coli* characteristics were sent to Pasteur Institute of Iran. For identification of *E. coli* strains, samples were cultured on MacConkey agar (Merck, Catalog No. 105465) and after O/N incubation (Incubator: Vision Korea), results were checked. For subsequent process, from each plate, five colonies were selected and were cultured on triple sugar iron agar media (TSI) (Merck, Catalog No. 104728) and after O/N incubation, strains with *E. coli* characteristics were selected. After this step, two colonies from five tubes which they were for one sample, selected and cultured in SIM medium (Merck, Catalog No. 105470), Simmons citrate (Merck, Catalog No.102501) and MRVP broth (Merck, Catalog No. 105712). Indol positive (Kovacs, Merck, Catalog No. 109293), MR positive (Methyl red, Merck, Catalog No. 106076), VP negative (KOH, SIGMA USA, Catalog No. P5958 and alpha naphthol-1 Catalog No. 70480) and citrate negative samples were identified as *E. coli.* All these selected samples were cultured in LB (Merck, Catalog No. 110285) and after O/N incubation, samples were centrifuged (Eppendorf Germany) and then they were stored at −70°C until used.

**Table 1.** Inclusion and exclusion criteria

**Inclusion Criteria**	**Exclusion Criteria**
Acute watery or bloody diarrhea	Chronic diarrhea
Patients with Iranian nationality	Traveler’s
Resident of selected provinces	Antibiotic consumption
	Immunodeficiency and chemotherapy
	Infants

### DNA extraction

For extracting DNA, genomes were purified by phenol-chloroform protocol (13, 14). SET solution [Sucrose 50mM (Merck. Catalog No. 107651), EDTA 10mM (SIGMA, Catalog No.03690), Tris-HCL pH 8.0 (Merck, Catalog No. 20-160)], NaOH 0.2N (Merck, Catalog No. 109136) and SDS 1% (SIGMA USA, Catalog No. L3771), KAC 5M (Merck, Catalog No. 168306), 200μl phenol (SIGMA, Catalog No. P1037) and 200μl chloroform (Merck, Catalog No. 107024) were used and after centrifuge, supernatant were collected. 1ml ethanol 96% and 1ml ethanol 70% were added and the samples were air dried. Appropriate amount of TE (10μ)- Rnase (5μl20μg/μl) was used and samples were stored at −20°C till used.

### PCR

All the genomes were checked by PCR assay. *lt* and *sta* genes were identified for ETEC, *eae* gene for atypical and *eae* and *bfp* for typical EPEC, *aa* gene for EAEC, *stx_1_* and *stx_2_* and *eae* genes for EHEC (*stx_1_* or *stx_2_* or both for STEC) and *invE* for EIEC. For preparation samples, 10μl Master Mix 2X (Fermentas, Catalog No. K0171), 7μl DDW, 1μl reverse and 1μl forward primers and at last 1μl sample were added. 1kb DNA ladder and ladder mix for positive controls and K12 for negative control were used. PCR program consist of denaturation, annealing and extension steps was performed by Eppendorf thermo cycler (Germany). Control strains employed in every PCR reaction were ETEC H10407, EPEC e2348/69, EHEC O157:H7, EAEC O42 and *Shigella* 12022 for EIEC. All the samples were checked by electrophoresis gel method and visualized by gel-documentation system.

## RESULTS

In this study, 170 samples from East-Azerbaijan, Gilan, Kurdistan, Tehran, Hamedan and Zanjan that were from different ages of males and females were evaluated. Samples were from winter and spring seasons. In the first step, 25 samples identified as non-*E. coli* and discarded. 145 (85%) samples identified as *E. coli* and their genomes extract by phenol-chloroform method and then checked by PCR assay. Finally identified as ETEC (n=10, 5.88%), EPEC (n=18, 10.58%), (n=6 3.52%) EHEC and STEC (n=12 7.05%). 22 (22.74=29.72%) positive samples were from winter and 24 (24/96=25%) positive samples were from spring season. None of the samples were positive for EAEC and EIEC. The positive PCR results were found for *lt* (n=5, 11.90 %), *sta* (n=4, 9.52 %), *lt* and *sta* (n=1, 2.38 %), *eae* (n=17, 40.47 %), *eae* and *bfp* (n=1, 2.38 %), *stx_1_* (n=3, 7.14%), *stx_2_* (n=3, 7.14 %), *stx_1_* and *stx_2_* (n=2, 4.76 %), *stx_1_* and *stx_2_* and *eae* (n=6, 14.28 %). Therefore, *eae* was the most prevalent gene in this study.

## DISCUSSION

This study was focused on *E. coli* pathotypes which causes diarrhea hence samples were collected from different regions. Different ages from children to adult and distribution of samples in 6 provinces were included in this study. Of 92 (54%) samples from males and 78 (46%) samples from females, 21 (22%) from females and 25 (32%) from males were identified as pathogenic *E. coli*. Children under 12 years old yeilded 16 positive samples (%34). Also positive samples of ETEC, EPEC, EHEC and STEC strains were isolated in both male and female. The most prevalent gene in this study was *eae* 17 (40.47 %). Salmanzadeh et al. from July to December 2003, investigated four categories of diarrheagenic *E. coli* in children with acute diarrhea. Stool specimens of children under 5 years of age with diarrhea (n=200) and matched controls (n=200) without diarrhea were studied for the presence of six different genes of DEC by PCR. STEC isolates were typed by O157 and H7 anti sera. EAEC was the most prevalent category and was found among 24% of patients with diarrhea and 8% of controls. ETEC was isolated from 15.5% of patients with diarrhea but not from any controls, STEC from 15% of patients and 2% of controls and EPEC from 6% of patients and 5% of controls. Of 30 STEC isolates from patients with diarrhea, seven were O157:H7 and 23 were non-O157:H7 (15). Alikhani et al. in 2013 conducted a study on DEC isolated from adolescents and adults in Hamedan, Western Iran. Among the 187 patients, 40 (21.4%) were positive for DEC. The most frequently identified DEC was EPEC (47.5%), followed by EAEC (20%), ETEC (17.5%) and STEC (15%). No isolates of EIEC were detected in their study, wich is similar to our finding. All STEC strains were *stx+* / *eaeA*−. Out of the 7 ETEC strains, 5 (71.4%) produced *ST*, one (14.3%) produced only *ST* and one of the isolates (14.3%) produced both *ST* and *LT* encoded by *est* and *elt* genes, respectively (8). Salmani et al. in 2016, studied on 200 stool samples from patients with diarrhea which were referred to Milad (Tehran, Iran) and Tohid (Sanandaj, Iran) hospitals. Prevalence of the genes encoding virulence factors for DEC were 62%, 25%, 24%, 13%, 7% and 5% for *ST* (ETEC), *LT* (ETEC), *aggR* (EAEC), *daaD* (DAEC), *invE* (EIEC) and *eae* (EPEC), respectively. ETEC and EAEC were the most detected *E. coli* among stool samples in this survey (3). Duta et al. in India, collected stool samples during 2008-2011 to analyze the trends in the prevalence of different pathogroups of DEC among hospitalized acute diarrheal patients. Samples were from children to adults. Multiplex PCR assay showed that the prevalence of EAEC was most common (5.7%) followed by ETEC (4.2%) and EPEC (1.8%). This study involved different ages and focus on *E. coli* like current study but the most prevalent gene was *aa* 218 (5.7%) (16). Karamb et al. in Kenya between March and July 2012 studied bacterial pathogens in children from 308 samples. A cross sectional study was conducted in Igembe District Hospital in Meru County to determine the burden and factors associated enteric bacterial infection among children aged five years and below. The bacterial isolation rates were ETEC 9.1%, EPEC 6.8% and EAEC 12.3%, *Salmonella* paratyphoid (10.4%), *Shigella flexineri* (1.9%) and *Shigella dysentriae* (0.9%) (17). Using PCR, Taiochi et al. studied the 147 samples from infants with diarrhea in Bangladesh in 2013 and detecte EAEC (12.1%), EPEC (10.2%), *Campylobacter, Entamoeba histolytica* and rotavirus as the most frequent probable contributors to diarrhea. The positive percentage of ETEC was 6.8% and the most prevalent gene was *eae,* similar to this study (18).

**Table 2.** PCR Primers

**Target**	**Forward**	**Reverse**	**Product size (bp)**
*elt*	GAACAGGAGGTTTCTGCGTTAGGTG	CTTTCAATGGCTTTTTTTTGGGAGTC	655
*estla*	CCTCTTTTAGYCAGACARCTGAATCASTTG	CAGGCAGGATTACAACAAAGTTCACAG	157
*invE*	CGATAGATGGCGAGAAATTATATCCCG	CGATCAAGAATCCCTAACAGAAGAATCAC	766
*aa*	CTGGCGAAAGACTGTATCAT	CAATGTATAGAAATCCGCTGTT	629
*eae*	TCAATGCAGTTCCGTTATCAGTT	GTAAAGTCCGTTACCCCAACCTG	482
*bfp*	GACACCTCATTGCTGAAGTCG	CCAGAACACCTCCGTTATGC	910
*stx_1_*	CGATGTTACGGTTTGTTACTGTGACAGC	AATGCCACGCTTCCCAGAATTG	244
*stx_2_*	GTTTTGACCATCTTCGTCTGATTATTGAG	AGCGTAAGGCTTCTGCTGTGAC	324

**Table 3.** Result of different positive samples in selected provinces

**Provinces**	**Total samples**	**Non- E. coli**	**ETEC**	**EPEC**	**EAEC**	**EHEC**	**STEC**	**EIEC**
East-Azerbaijan	12 (12/170=7.50%)	3 (3/170=1.76%)	2 (2/170=1.17%)	2 (2/170=1.17%)	_	_	_	_
Gilan	20 (20/170=11.76%)	1 (1/170=0.58%)	3 (3/170=1.76%)	1 (1/170=0.58%)	_	_	1 (1/170=0.58%)	_
Kurdistan	46 (46/170=27.05%)	8 (8/170=4.70%)	_	3 (3/170=1.76%)	_	_	3 (3/170=1.76%)	_
Tehran	35 (35/170=20.58%)	6 (6/170=3.52%)	2 (2/170=1/17%)	4 (4/170=2.35%)	_	2 (2/170=1.17%)	5 (5/170=2.94%)	_
Hamedan	42 (42/170=24.70%)	5 (5/170=2.94%)	3 (3/170=1.76%)	4 (4/170=2.35%)	_	2 (2/170=1.17%)	1 (1/170=0.58%)	_
Zanjan	15 (15/170=8.82%)	2 (2/170=1.17%)	_	4 (4/170=2.35%)	_	2 (2/170=1.17%)	2 (2/170=1.17%)	_
Total	170	25 (14.70%)	10 (6.89%)	18 (12.14%)	_	6 (4.13%)	12 (8.27%)	_

This study involved samples from north and northwest provinces of Iran. Complementary studies like serotyping, clonality and antibiogram susceptibility testing for these samples could provide additional data. Moreover a study covering whole year and in other region of the country can help to adopt necessary policies against possible outbreaks in Iran.
